# In Vitro Investigation of Pulsed Electromagnetic Field Stimulation (PEMF) with MAGCELL^®^ ARTHRO on the Regulatory Expression of Soluble and Membrane-Bound Complement Factors and Inflammatory Cytokines in Immortalized Synovial Fibroblasts

**DOI:** 10.3390/jpm14070701

**Published:** 2024-06-29

**Authors:** Sandeep Silawal, Markus Gesslein, Maximilian Willauschus, Gundula Schulze-Tanzil

**Affiliations:** 1 Institute of Anatomy and Cell Biology, Paracelsus Medical University, Nuremberg and Salzburg, General Hospital Nuremberg, Prof. Ernst Nathan Str. 1, 90419 Nuremberg, Germany; gundula.schulze@pmu.ac.at; 2 Department of Orthopedics and Traumatology, Paracelsus Medical University, Nuremberg and Salzburg, General Hospital Nuremberg, Breslauer Str. 201, 90471 Nuremberg, Germany; markus.gesslein@klinikum-nuernberg.de (M.G.); mp-willauschus@outlook.com (M.W.)

**Keywords:** cytokines, complement regulatory proteins, osteoarthritis, PEMF, synovial fibroblasts, synovitis

## Abstract

Pulsed electromagnetic field stimulation (PEMF) is gaining more attention as a non-invasive arthritis treatment. In our study, immortalized synovial fibroblasts (K4IM) derived from a non-arthritic donor were exposed to MAGCELL^®^ ARTHRO, a PEMF device, with 105 mT intensity, 8 Hz frequency, and 2 × 2.5 min sessions conducted thrice with a 1 h interval, to understand the underlying mechanism in regard to the complement system. Additionally, tumor necrosis factor (TNFα, 10 ng/mL) pre-treatment prior to PEMF stimulation, as well as 3-day versus 6-day stimulation, were compared. Gene expression of C4b binding protein-alpha and -beta (C4BPα, C4BPβ), complement factor (CF)-H, CFI, CD55, CD59, Interleukin (IL-6) and TNFα was analyzed. Immunofluorescence staining of CD55, CD59, and Ki67 was conducted. Results showed the absence of C4BPα gene expression, but C4BPβ was present. One and three days of PEMF stimulation caused no significant changes. However, after six days, there was a significant increase in CD55, CFH, and CD59 gene expression, indicating cytoprotective effects. Conversely, IL-6 gene expression increased after six days of stimulation and even after a single session in TNFα pre-stimulated cells, indicating a pro-inflammatory effect. PEMF’s ambivalent, i.e., enhancing complement regulatory proteins and pro-inflammatory cytokines, highlights its complexity at the molecular level.

## 1. Introduction

Osteoarthritis (OA) stands as the most prevalent degenerative joint disorder globally and affects more than 500 million population worldwide [1,2]. While age is a primary factor in its occurrence, factors such as obesity, mechanical misalignment, joint instability, and genetic predisposition also significantly contribute to OA [3]. Consequently, the increasing prevalence of OA within our aging population, coupled with rising comorbidities, may substantially augment the future economic and social burdens on society. A profound comprehension of OA pathogenesis is crucial for managing the processes of cartilage depletion that lead to joint destruction.

The contemporary understanding of OA pathogenesis emphasizes its involvement across multiple tissues [4]. Hence, a comprehensive investigation into each tissue component—cartilage, subchondral bone, and synovium is necessary [5]. Synovial inflammation of varying degrees, alongside hypertrophy of the joint capsule, emerges as a principal pathological feature in OA-afflicted joints [5]. In addition to the degradation of the articular cartilage, various other pathologies such as thickening of the subchondral bone, osteophyte formation, degeneration of ligaments and menisci, inflammation of the synovium and thickening of the joint capsule are observed in the OA pathogenesis [5,6].

OA therapy poses significant clinical challenges [7]. The presence of comorbidities, the need for chronic pain management, limited functionality due to steady disease progression, and the often less optimistic outlook for improvement in patients all necessitate extensive clinical and psychological support for patients [7,8]. Current conservative pharmacological management strategies aim to alleviate pain using topical or oral nonsteroidal anti-inflammatory drugs (NSAID) and opioids for intense pain, alongside other measures such as capsaicin, antidepressants such as Duloxetine, and intra-articular anti-inflammatory agents such as glucocorticoids or hyaluronic acid. Maqbool et al., 2021 have briefly discussed the clinical prospects and management of OA [9]. Consequently, as conservative therapies falter, advanced OA joints often necessitate surgical intervention for joint replacement to restore function and alleviate pain [9]. Given the limitations and potential side effects of prolonged NSAID use, there is a pressing need for alternative approaches to OA therapy.

One promising adjunctive therapy is pulsed electromagnetic field (PEMF) therapy, which has been viewed as a potential candidate for alleviating joint pain and improving function [10,11,12,13]. However, critical reviews also suggest mixed results regarding its efficacy in treating joint pain and stiffness, though it may enhance physical function compared to placebo [14,15]. The MAGCELL^®^ ARTHRO device, transmitting a sinusoidal waveform of strong PEMF at 105 mT with a frequency of 8 Hz, has garnered attention for its reported pain reduction and increased mobility in OA patients. A prospective, placebo-controlled, double-blind study involving 57 knee OA patients demonstrated significant reductions in pain, joint stiffness, and improved functionality with the use of this device [16]. Similarly, Bagnato et al., in a double-blind, placebo-controlled, randomized clinical trial, showed that PEMF therapy improved pain and dysfunction in patients with knee OA [12].

Despite promising clinical outcomes, the molecular mechanisms underlying PEMF therapy in joint tissues still need to be studied in detail. The complement system, a key component of the immune system, plays a crucial role in orchestrating inflammatory responses locally and systemically [4,17]. The complement system is typically activated through various pathways: the classical pathway, initiated by C1q binding directly to pathogen surfaces or antibody–antigen complexes; the lectin pathway, triggered by lectin binding to mannose-containing carbohydrates on pathogens; and the alternative pathway, initiated by spontaneous hydrolysis of complement protein C3 (Supplementary Materials) [17]. Zymogen precursors are cleaved into fragments, forming active enzymes that further split subsequent zymogens in the complement pathway, ultimately leading to cell lysis via the membrane attack complex [17]. During complement activation, anaphylatoxins such as C4a, C3a, and C5a are generated through the cleavage of proteins C4, C3, and C5, respectively, playing active roles in the inflammatory process [17]. Soluble and membrane-bound complement regulatory proteins (CrP) help regulate hyperinflammation, mitigating the detrimental effects of complement activity [18].

Complement factor I (CFI), predominantly synthesized in the liver, is an 88 kDa plasma protein consisting of two disulfide-linked chains: the α-chain (50 kDa) and the β-chain (38 kDa). Acting as a serine protease, CFI cleaves the α-chains of C4b and C3b with the assistance of cofactors such as complement factor H (CFH) and C4b binding protein (C4BP) [19]. CFI concentration increases during inflammation, with normal serum or plasma concentrations ranging between 19 and 64 µg/mL [19]. C4BP, primarily produced by hepatocytes, consists of seven thin, elongated subunits (α-chain, 70 kDa) linked to a central body (β-chain, 45 kDa), functioning as a cofactor for CFI [20]. With approximately 150 µg/mL plasma concentration, C4BP functions as mentioned before as a cofactor for CFI, which undergoes complement regulatory function interfering with the assembly of the C3- and C5-convertases, as well as contributing to the disassembly of the C3-convertase [20]. CFH (43 kDa) is an abundant serum glycoprotein produced primarily in the hepatocytes [21]. The serum concentration can vary widely between 116 and 562 µg/mL as summarized in a review paper [22]. CFH plays a crucial role in accelerating the disassembly of the alternative pathway C3 convertase (C3bBb) and acts as a cofactor for CFI-mediated cleavage of C3b [22]. CD55 (43 kDa) and CD59 (18–20 kDa) are membrane-bound CrP that regulate complement activation at different cascade levels. CD55 accelerates C3 convertase degradation at the intermediate level, while CD59 inhibits membrane attack complex assembly [17].

Some research has described the anti-inflammatory properties of PEMF stimulation at the molecular level, such as decreasing IL1-ß or TNFα [23,24]. This study aimed to further investigate the anti-inflammatory properties of PEMF treatment, specifically its direct influence on complement regulation in synovial fibroblasts, which has never been studied before.

## 2. Materials and Methods

### 2.1. In Vitro Culture of K4IM

The SV40 T-antigen immortalized synovial fibroblast cell line, K4IM, was generously provided by Dr. Christian Kaps. These cells were cultured in a growth medium supplemented with 10% fetal bovine serum (FBS) (PAN-Biotech, Aidenbach, Germany), which also served as Ctrl Medium 1. The growth medium was composed of Dulbecco’s MEM/Ham’s F-12 (1:1) supplemented with 25 mg/mL ascorbic acid, 50 IU/mL streptomycin, 50 IU/mL penicillin, 2.5 μg/mL amphotericin B, and essential amino acids (all products from Carl Roth GmbH, Karlsruhe, Germany). For cell passaging, 0.05% trypsin/1.0 mM EDTA (Carl Roth GmbH) was utilized before seeding the cells for stimulation.

### 2.2. PEMF Stimulation of the Synovial Fibroblasts

**Figure 1:**
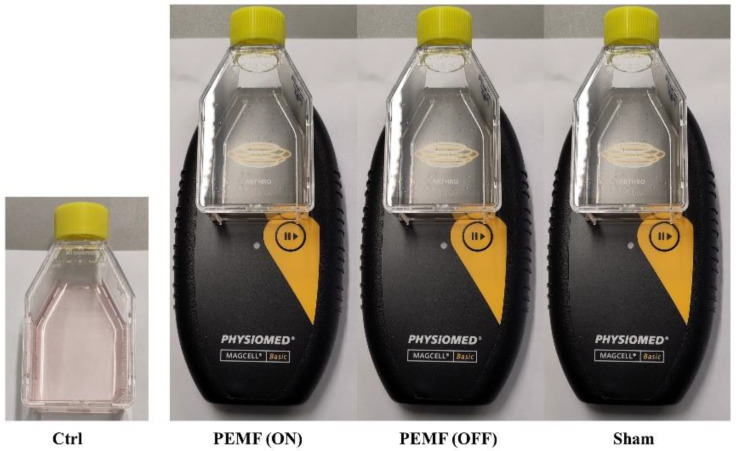
Experimental setting for Study 1 (Table 1—Day 3). K4IM synovial fibroblasts in Ctrl Medium 2 stimulated with PEMF (ON), PEMF (OFF) or sham and non-stimulated group as control (Ctrl). Each stimulation session comprised an application time of 2 × 2.5 min 3 times with an interval of 1 h between each stimulation.

Study 1: K4IM synovial fibroblasts were seeded at a density of 10,000/cm^2^ either in T25 culture flasks (Sarstedt, Nümbrecht, Germany) or on glass coverslips, depending on the experiment, and cultured in Ctrl Medium 1 (3 mL/flask in T25 flasks). After 48 h, the supernatant was aspirated, and the cells were washed with PBS before being supplied with 1% FBS-supplemented growth medium (Ctrl Medium 2) (2 mL/flask). Following a 1 h incubation, the supernatant was replaced by fresh Ctrl Medium 2, and the stimulation commenced (Figure 1, Table 1).

The stimulation involved exposing the K4IM cells in the flasks to the anterior surface of the MAGCELL^®^ ARTHRO apparatus (Catalog #AC-1815404, Physiomed Elektromedizin AG, Schnaittach, Germany) for 5 min. As a test, metal powder, distributed homogeneously on the floor of the T25 cell culture flask was treated with the apparatus. The activation of the apparatus showed that the electromagnetic influence could be seen up to the periphery of the flask (Supplementary Materials). As described in a 2015 clinical study, the MAGCELL^®^ ARTHRO device comprises rotating magnets [16]. It utilizes time-varying magnetic fields with a flux density of 105 mT, providing current densities exceeding 10 mA/cm^2^. During stimulation, the side of the culture flask with adherent cells (inferior side/floor) was positioned against the anterior surface of the apparatus. The protocol for a single-day stimulation session involved an application time of 2 × 2.5 min for 3 sessions with a 1 h interval between each stimulation. In the aforementioned clinical study, the placebo group received a similar device with an identical exterior, but non-magnetic rotating segments [16]. In our study, we referred to the stimulation group as sham. Five independent experiments (n = 5) were performed in Study 1.

Study 2: Similar to Study 1, K4IM synovial fibroblasts were seeded and cultured for the experiment in Ctrl Medium 1 (2 mL/flask in T25 flasks). After 24 h, the supernatant was aspirated, and the cells were washed with PBS. Group 1 and Group 2 received Ctrl Medium 1, while Group 3 and Group 4 were pre-stimulated with recombinant TNFα (Peprotech, Princeton, NJ, USA) at a final concentration of 10 ng/mL diluted in Ctrl Medium 1 (2 mL/flask). On day 3, following a 1 h starvation period with Ctrl Medium 2, the medium was replaced (2 mL/flask), and PEMF stimulation commenced, as described previously, with 3 sessions spaced 1 h apart for the stimulation groups (see Table 2). Five independent experiments (n = 5) were performed in Study 2.

Study 3: K4IM synovial fibroblasts were seeded at a density of 5000/cm^2^ and cultured in Ctrl Medium 1 (2 mL/flask in T25 flasks) for the experiment. After 24 h, the supernatant was aspirated, and the cells were washed with PBS before being supplied with Ctrl Medium 2 (2 mL/flask). Following a 1 h starvation period, the medium was replaced (2 mL/flask), and PEMF stimulation commenced, as previously described, with 3 sessions spaced 1 h apart for the stimulation groups. Groups 1–2 underwent stimulation for 3 days, while groups 3–4 were stimulated for a total of 6 days (see Table 3). Starting from day 3, 50% of the medium was replaced with fresh medium daily. Four independent experiments (n = 4) were performed in Study 3.

### 2.3. Gene Expression Analysis

#### 2.3.1. RNA Isolation and cDNA Synthesis

Synovial fibroblasts were seeded as described in Section 2.2. Post-stimulation, these cells were washed with PBS. Lysis of the cells was achieved using a 1:100 solution of β-Mercaptoethanol (Sigma-Aldrich, St. Louis, MO, USA) in RNeasy Lysis Buffer (Qiagen, Hilden, Germany). Total RNA extraction was performed using the RNeasy Mini Kit (Qiagen) following the manufacturer’s protocol [25]. The RNA quantity was assessed using the NanoDropTM 2000 Spectrophotometer (Thermo Fischer Scientific, Erlangen, Germany). Subsequently, cDNA was transcribed using the QuantiTect Transcription Kit (Qiagen) with Mastercycler^®^ (Eppendorf, Hamburg, Germany) according to the manufacturer’s instructions.

#### 2.3.2. qPCR

For real-time polymerase chain reaction (qPCR) analyses, glyceraldehyde-3-phosphate dehydrogenase (GAPDH) served as the reference gene, and specific primers from TaqMan^®^ Gene Expression Assays (Applied Biosystems, Foster City, CA, USA), listed in Table 4, were utilized. Each well contained 20 ng of cDNA template (1 µL), TaqMan^®^ Gene Expression Master Mix (Applied Biosystems), and water (9 µL) to perform the TaqMan^®^ Gene Expression Assay [25]. Standard TaqMan^®^ analysis protocol as provided by the manufacturer was followed, conducted in the Applied Biosystems StepOnePlus^TM^ Real-Time PCR System. Relative gene expression levels were determined using the delta–delta Ct method, normalized against the reference gene [26].

### 2.4. Protein Expression Analysis

#### Immunofluorescence Labeling of the Synovial Fibroblasts

Following PEMF stimulation, K4IM cells were washed with Tris-buffered saline (TBS) (Medicago AB, Uppsala, Sweden) and fixated with a 4% paraformaldehyde solution (PFA) (Morphisto, Frankfurt, Germany) for 15 min. The staining protocol has been detailed in our prior publication [27]. Primary antibodies utilized are listed in Table 5. Cell nuclei were counterstained using 4′,6-diamidino-2-phenylindole (DAPI) (Roche Diagnostics GmbH, Basel, Switzerland), while the cytoskeleton was stained with Phalloidin Alexa Fluor 633 (ThermoFisher Scientific). Images of immunolabeled cells were captured using confocal laser scanning microscopy (SPE-II, Leica Microsystems, Weztlar, Germany). Total protein intensity per cell (quantified using DAPI) was measured in each group for analysis.

### 2.5. Statistical Analysis

Statistical analyses were conducted using GraphPad Prism (Version 8.1.4) from GraphPad Software, San Diego, CA, USA. The Shapiro–Wilk normality test was performed, and normalized data were presented as mean ± standard deviation (mean ± SD). Significance between experimental groups was determined at *p* < 0.05 using one-way ANOVA analysis with Tukey’s multiple comparisons test. The Grubbs test was utilized to identify and exclude outliers from the statistical analysis.

## 3. Results

### 3.1. Gene Expression

C4BPα gene expression could not be detected in the immortalized synovial fibroblasts. However, the C4BPβ gene was expressed in these cells.

**Figure 2:**
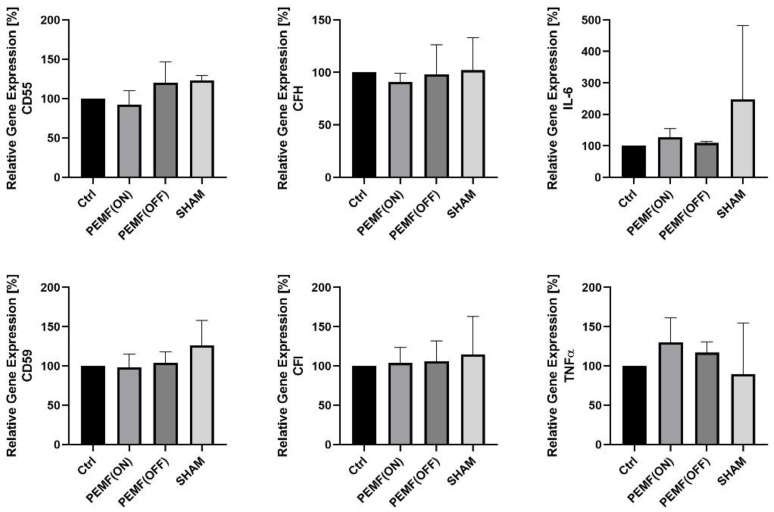
Graphic representation of relative gene expression of complement factors (CD55, CD59, CFH and CFI) and cytokines (IL-6 and TNFα) in synovial fibroblast cell line K4IM after PEMF stimulation [PEMF(ON)] compared to various controls. A one-day stimulation protocol was used. Ctrl = non-stimulated group, [PEMF(OFF)] = apparatus turned off, sham = apparatus turned on without electromagnetic impulse. n = 5 independent experiments. Mean with standard deviation (SD). Non-stimulated group as control has been normalized to 100. Repeated measures one-way ANOVA using Tukey’s multiple comparisons.

Study 1: In the provided setup (Table 1, Figure 1), synovial fibroblasts were exposed to MAGCELL^®^ ARTHRO stimulation for a single day. The stimulation group PEMF(ON) was compared with several controls: a non-stimulation group (Ctrl), a group with the apparatus turned off PEMF(OFF), and a sham group (Figure 1). However, the stimulation did not exert any significant influence on the gene expression of the analyzed proteins (Figure 2).

**Figure 3:**
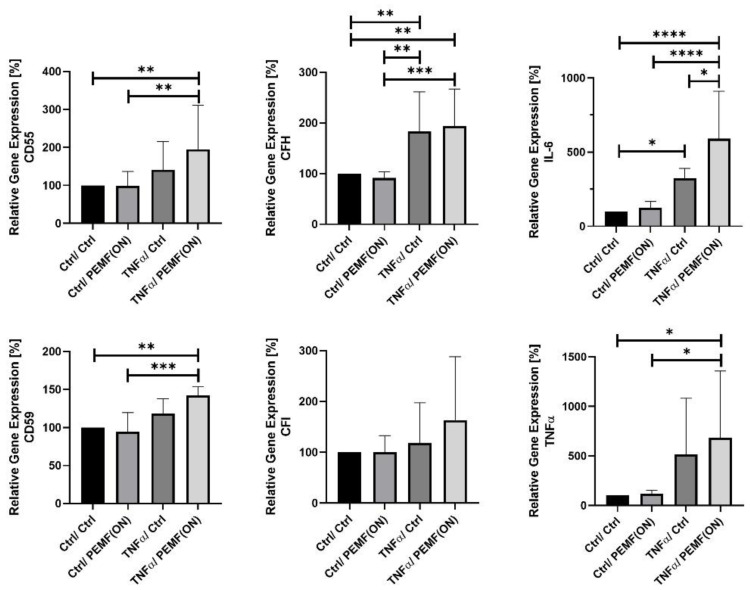
Graphic representation of relative gene expression of complement factors (CD55, CD59, CFH and CFI) and cytokines (IL-6 and TNFα) in synovial fibroblast cell line K4IM after PEMF stimulation [PEMF(ON)] with and without 24 h TNFα pre-stimulation (10 ng/mL) compared to their respective control (ctrl). A one-day stimulation protocol was used. Ctrl = non-stimulation. n = 5 independent experiments. Mean with standard deviation (SD). Non-stimulated group without TNFα pre-stimulation (Ctrl/Ctrl) has been normalized to 100. Repeated measures one-way ANOVA using Tukey’s multiple comparisons (*). * = *p* ≤ 0.05, ** = *p* ≤ 0.01, *** = *p* ≤ 0.001, **** = *p* ≤ 0.0001.

Study 2: In this inflammation model (Table 2), synovial fibroblasts were pre-stimulated with TNFα for 24 h before PEMF stimulation PEMF(ON). Similar to Study 1, a single treatment session without TNFα pre-stimulation did not alter the gene expression of analyzed proteins compared to the Ctrl/Ctrl group. TNFα stimulation alone, i.e., TNFα/Ctrl significantly elevated the gene expression of CFH and IL-6 in K4IM compared to the Ctrl/Ctrl group. However, TNFα pre-stimulated cells, when subjected to PEMF, i.e., TNFα/PEMF(ON), exhibited increased gene expression of IL-6. Both Ctrl/Ctrl and Ctrl/PEMF(ON) consistently displayed significantly lower gene expression in analyzed complement components, except for CFI, compared to TNFα/Ctrl and TNFα/PEMF(ON) groups, respectively (Figure 3).

**Figure 4:**
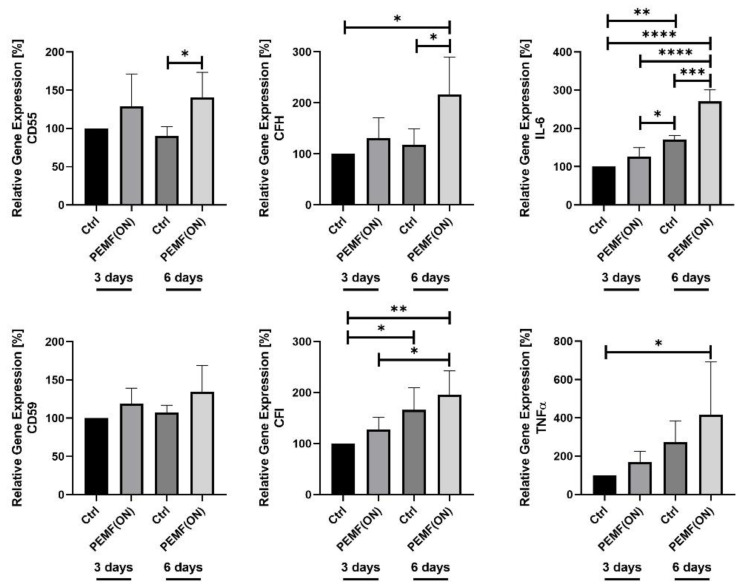
Graphic representation of relative gene expression of complement factors (CD55, CD59, CFH and CFI) and cytokines (IL-6 and TNFα) in synovial fibroblast cell line K4IM after PEMF stimulation [PEMF(ON)] for 3 days and 6 days compared to their respective control (ctrl). Ctrl = non-stimulation. n = 4 independent experiments. Mean with standard deviation (SD). Non-stimulated group (3 days) has been normalized to 100. Repeated measures one-way ANOVA using Tukey’s multiple comparisons (*) * = *p* ≤ 0.05, ** = *p* ≤ 0.01, *** = *p* ≤ 0.001, **** = *p* ≤ 0.0001.

Study 3: In this experimental setup (Table 3), the PEMF stimulation duration was extended to examine its long-term effects compared to previous studies. Thus, synovial fibroblasts were stimulated with PEMF(ON) for 3 days and 6 days, with respective non-stimulated groups serving as controls. After 3 days of stimulation, no significant changes were observed in the gene expression of the analyzed proteins compared to the non-stimulated control. However, there was a notable trend toward increased gene expression in all proteins. After 6 days of stimulation, there was a marked increase in gene expression. Specifically, CD55, CFH, and IL-6 gene expression showed significant elevation in the PEMF(ON) stimulated group compared to the non-stimulated control (Ctrl). Furthermore, the non-stimulated group (Ctrl) displayed significantly lower gene expression of CFI and IL-6 after 3 days compared to the same group after 6 days. Interestingly, the stimulated group PEMF(ON) after 3 days also exhibited lower expression of these genes compared to the same group after 6 days (Figure 4).

### 3.2. Protein Expression

**Figure 5:**
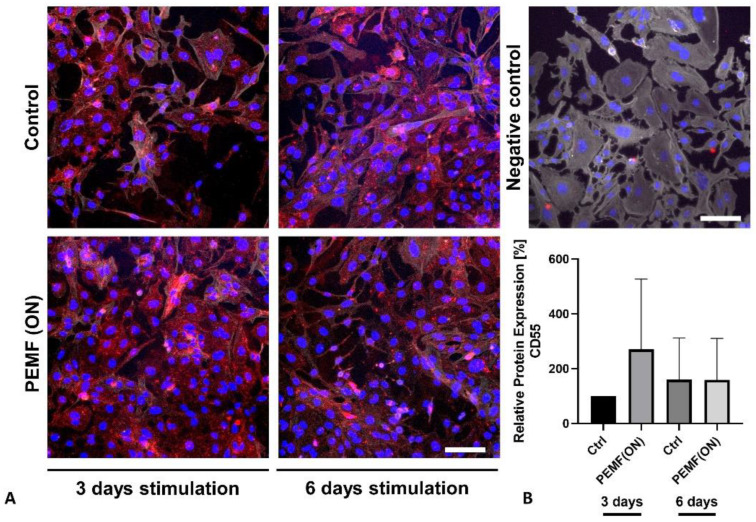
(**A**) Representative images of K4IM cell line after 3 days and 6 days PEMF(ON) stimulation compared to their non-stimulated counterparts, respectively (200× magnification), immunolabeled with a CD55 specific antibody and negative control of the staining. Red (Cy3) = CD55, blue (DAPI) = cell nuclei, gray (Phalloidin Alexa Fluor 633) = F-Actin cytoskeleton. Scale bar = 100 µm. (**B**) Graphic representation of relative CD55 protein fluorescence intensity, n = 4 independent experiments, mean with standard deviation (SD). Control (3 days) has been normalized to 100. Repeated measures one-way ANOVA using Tukey’s multiple comparisons.

**Figure 6:**
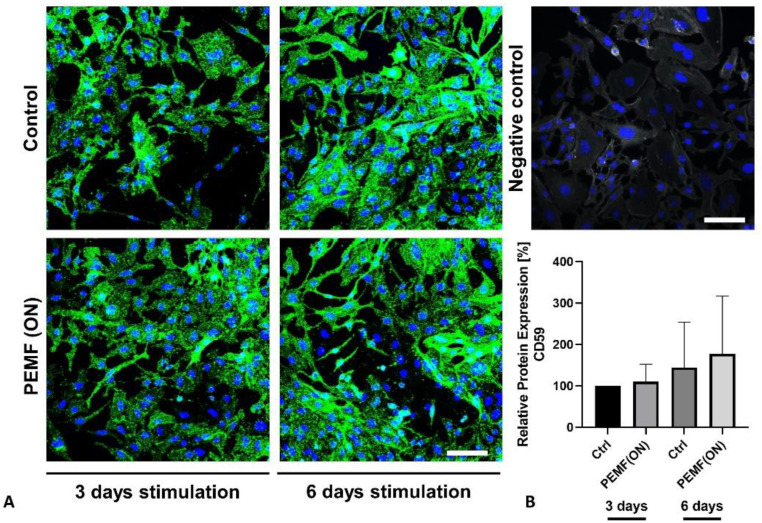
(**A**) Representative images of K4IM cell line after 3 days and 6 days PEMF(ON) stimulation compared to their non-stimulated counterparts, respectively (200× magnification), immunolabeled with a CD59 specific antibody and negative control of the staining. Green (Alexa Fluor 488) = CD59, blue (DAPI) = cell nuclei, gray (Phalloidin Alexa Fluor 633) = F-Actin cytoskeleton. Scale bar = 100 µm. (**B**) Graphic representation of relative CD59 protein fluorescence intensity, n = 4 independent experiments, mean with standard deviation (SD). Control (3 days) has been normalized to 100. Repeated measures one-way ANOVA using Tukey’s multiple comparisons.

In the analyzed synovial fibroblasts, both CD55 and CD59 protein expressions were observed. However, there were no significant changes noted after 3 days or 6 days of PEMF stimulation compared to their respective controls (Figure 5 and Figure 6). Interestingly, PEMF(ON) stimulation did show a tendency toward increased CD55 protein expression after 3 days (Figure 5), and a similar non-significant trend was seen for CD59 protein expression after 6 days of PEMF(ON) stimulation (Figure 6).

**Figure 7:**
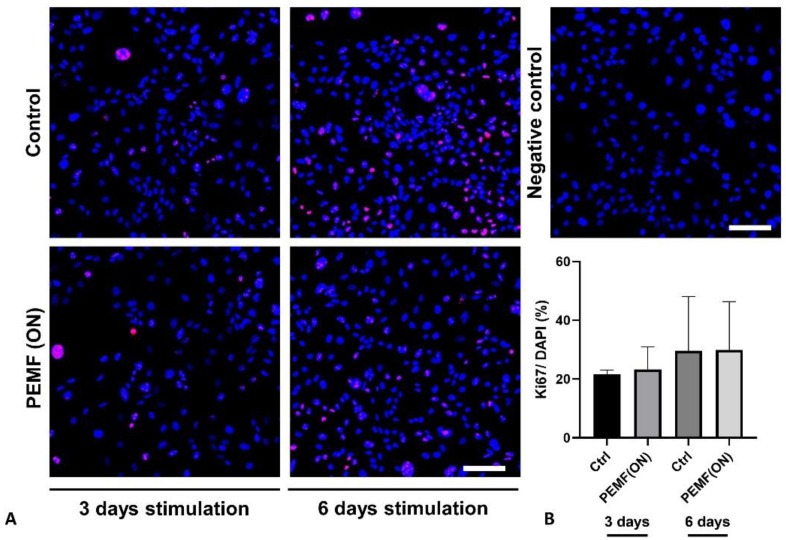
(**A**) Representative images of K4IM cell line after 3 days and 6 days PEMF(ON) stimulation compared to their non-stimulated counterparts, respectively (200× magnification), immunolabeled with Ki67 specific antibody and negative control of the staining. Red (Cy3) = Ki67, blue (DAPI) = cell nuclei. Scale bar = 100 µm. (**B**) Graphic representation of relative CD55 protein fluorescence intensity, n = 3 independent experiments, mean with standard deviation (SD). Control (3 days) has been normalized to 100. Repeated measures one-way ANOVA using Tukey’s multiple comparisons.

Also, Ki67 staining was performed (Figure 7). However, no significant differences were detected among the groups, although there was a slight inclination toward higher proliferative activity in the 6-day experimental groups. Additionally, staining with phalloidin to visualize the F-actin cytoskeleton revealed no notable differences in response to the treatment across the groups.

## 4. Discussion

In our study, utilizing K4IM cells derived from a non-arthritic donor, we observed the expression of the C4BPβ gene, while the expression of C4BPα was not detected. Similarly, Criado-García et al., (1999) found that fibroblast-like cells isolated from human ovaries expressed C4BPβ but not C4BPα at both, the gene and protein levels [28]. Another study examining arthritic joints revealed the presence of the C4BPβ peptide in the cytoplasm of lining cells and in most subintimal fibroblast-like cells within the tissue [29]. Immunohistochemistry signals and protein levels of C4BPβ were higher in synovial effusions from rheumatoid arthritis joints compared to osteoarthritic joints in that study. While C4BPα was not detected inside the cells, it was found in the interstitial areas of the subintimal region in many synovial tissues of patients with rheumatoid arthritis.

Study 1: This study aimed to investigate the impact of PEMF(ON) on the gene expression of K4IM cells compared to various control groups. In addition to non-stimulated cells (Ctrl), both, PEMF(OFF) and sham groups were included. The sham group involved cell stimulation using a non-electromagnetic apparatus, serving as a control in our experimental setup. We found that none of the groups exhibited any significant changes after a single-day session of PEMF stimulation. These findings suggest that brief exposure of K4IM cells to PEMF has minimal influence and does not yield statistically significant results in terms of gene expression. Therefore, a single session of PEMF stimulation is less likely to have any significant impact in practical use. 

Study 2: In an inflammation model, cells were pre-stimulated with TNFα for 24 h before a single PEMF stimulation session. Comparing TNFα pre-stimulation of K4IM cells with no PEMF stimulation to the control group, higher gene expression levels were observed for all analyzed proteins, with statistically significant results noted for CFH and IL-6 expression. Previous research demonstrated that 24 h stimulation of K4IM cells with TNFα led to an approximately 40-fold increase in TNFα gene expression [27]. Additionally, in the same study, IL-6 gene expression was nearly 8-fold higher in the non-stimulated synovial fibroblast cell line HSE, derived from arthritic joints, compared to K4IM cells [27]. A single PEMF treatment in TNFα pre-stimulated cells also resulted in almost a 2-fold increase in IL-6 gene expression compared to TNFα pre-treated cells without PEMF stimulation. This effect could imply that either a single session of PEMF stimulation exacerbates inflammation in the inflammation model, or the increase in IL-6 gene expression needs a new interpretation. This heightened IL-6 gene expression in the inflammation model highlights the multifaceted role of IL-6 in the immune system. Despite its classification as a pro-inflammatory cytokine, recent years have seen recognition of the anti-inflammatory actions of IL-6 [30]. The increase in IL-6 gene expression could potentially contribute to the clinical improvement of patients, as demonstrated by Wuschech et al., (2015) in a prospective, placebo-controlled, double-blind study. However, it is essential to acknowledge that the simplified cell culture model used in our study may not fully reflect the systemic in vivo anti-inflammatory effect induced by PEMF in patients [16]. Moreover, future research should explore longer time frames to understand the long-term effects of PEMF stimulation in the inflammation model.

Study 3: Orfei et al. conducted a pilot study using a rat tendinopathy model induced by injecting type I collagenase into the Achilles tendon, followed by exposure to PEMF stimulation [31]. The authors observed that longer PEMF stimulation resulted in improved collagen fiber organization, reduced cell density, vascularity, and fat deposition, and restoration of physiological cell morphology compared to untreated tendons. However, similar to the 1-day stimulation in studies 1 and 2, a 3-day stimulation period did not yield significant changes in gene expression compared to the non-stimulated control group. Nonetheless, there was a noticeable trend toward increased gene expression in the treatment groups as the stimulation period extended.

After 6 days of PEMF(ON) stimulation compared to the control group, significantly higher expression of CD55, CFH, and IL-6 genes was observed. This trend was also reflected in semi-quantitative analysis of immunofluorescence-stained images, where a stronger CD55 signal was detected in the PEMF-stimulated group compared to the non-stimulated group. Karpus et al., (2015) found abundant CD55 expression in fibroblast-like synovial fibroblasts of the intimal lining layer, associated with linearly oriented reticular fibers, which were resistant to phospholipase C treatment [32]. This suggests a protective role of CD55 in synovial tissue against immune complex-mediated arthritis. Consistent with our previous research, CD55 gene expression was nearly 4-fold lower in arthritis-derived HSE synovial fibroblasts compared to K4IM cells, further highlighting the protective role of PEMF stimulation [27]. CFH and its variant, Factor H-like protein 1, may also play a protective role against complement damage in the synovium [33]. PEMF stimulation in K4IM cells induced nearly a 2-fold increase in CFH gene expression, suggesting enhanced regulation of complement activation in synovial fibroblasts. 

In terms of cytokine gene expression, Zou et al., (2017) demonstrated that IL-1β and TNFα levels secreted by nucleus pulposus cells into the culture medium were significantly reduced following PEMF exposure in an intensity-dependent manner [24]. Similarly, another study reported a reduction in IL-1β and TNFα secretion in fibroblast-like cells derived from mononuclear peripheral blood on days 14 and 21 of culture, along with an induction of IL-10 on day 21 of culture [23]. However, this study also found that PEMF did not inhibit the production of the cytokines IL-6 and IL-8. Our study revealed a significant increase in IL-6 gene expression after 6 days of PEMF stimulation compared to the non-stimulated group. This effect was observed even after a single session of stimulation, but only when the cells were pre-stimulated with TNFα. Interestingly, the group stimulated with PEMF for 6 days showed higher expression of CFI and IL-6 genes compared to the same group stimulated for only 3 days. This change in gene expression of CFI and IL-6 was also observed comparing the control groups of the two time periods, suggesting that cell proliferation or cell density may influence CFI and IL-6 gene expression, but not the expression of the other analyzed complement genes CD55, CD59, and CFH.

Regarding cellular response, PEMF stimulation has been shown to increase cell proliferation in osteoblasts and chondrocytes, cell migration as well as enhance extracellular matrix production [34,35,36,37]. However, in our study, we did not observe a significant increase in proliferative activity in K4IM synovial fibroblasts following PEMF stimulation for 3 days or 6 days. Even a longer stimulation time period should be analyzed in the future.

## 5. Limitations of the Study

The PEMF stimulation duration in a single session, the number of stimulation days, the stimulation intensity, and various other biological and non-biological factors could have varying impacts at the molecular level of the cells. One of the limitations of our study is the use of an immortalized cell line cultured under monolayer conditions. We recommend that future studies should be performed using different primary cell types freshly isolated from joint tissues at various time points, in a three-dimensional or co-culture model, to gain a more comprehensive understanding of PEMF stimulation effects on the entire joint. Although novel data were acquired through our study, incorporating other quantitative and semi-quantitative assays such as Western blots will be necessary to gain a broader picture of the response to PEMF stimulation in the future.

Our immune system is very complex because it requires a balanced hemostasis of soluble as well as membrane-bound pro- and anti-inflammatory components, such as cytokines and complement factors. The focus of this study was limited to two soluble complement factors (CFH and CFI), two membrane-bound complement factors (CD55 and CD59), and two pro-inflammatory cytokines (TNFα and IL-6). The overall complexity of the interplay between numerous complement factors and immune regulatory cytokines (such as IL-1, IL-33, IL-37, IL-10, etc.) needs further investigation. In addition to the production of these components, the effects of PEMF stimulation on phenotype, cell proliferation, and migration should be studied thoroughly in future research. This is also a limitation of our study.

## 6. Conclusions

Previous clinical studies have demonstrated the efficacy of PEMF therapy in alleviating osteoarthritic symptoms. However, both short-term (single-day) and three-day sessions of PEMF stimulation of K4IM synovial fibroblasts failed to induce significant changes in the gene expression of complement regulatory proteins (CD55, CD59, CFH, CFI) and cytokines (IL-6, TNFα) compared to six-day stimulation. This implies the importance of longer PEMF stimulation in practical applications. Nevertheless, a notable increase in the gene expression of CD55, CFH, and CD59 suggests that PEMF stimulation may confer cytoprotective and anti-inflammatory properties. Conversely, the upregulation of IL-6 gene expression indicates a pro-inflammatory response to PEMF stimulation. This dualistic effect underscores the complexity of PEMF’s impact on cells at the molecular level. Further research is necessary to deepen our understanding of PEMF’s influence on complement activity and cytokine regulation.

## Figures and Tables

**Table 1:** Stimulation groups and schedule for Study 1. Ctrl Medium 1 = 10% fetal bovine serum supplemented growth medium. Ctrl Medium 2 = 1% fetal bovine serum supplemented growth medium.

	Group 1	Group 2	Group 3	Group 4
Day 1	Ctrl Medium 1	Ctrl Medium 1	Ctrl Medium 1	Ctrl Medium 1
Day 2	Ctrl Medium 1	Ctrl Medium 1	Ctrl Medium 1	Ctrl Medium 1
Day 3	Ctrl Medium 2	PEMF (ON)	PEMF (OFF)	Sham

**Table 2:** Stimulation groups and schedule for Study 2. Ctrl Medium 1 = 10% fetal bovine serum supplemented growth medium. Ctrl Medium 2 = 1% fetal bovine serum supplemented growth medium.

	Group 1	Group 2	Group 3	Group 4
Day 1	Ctrl Medium 1	Ctrl Medium 1	Ctrl Medium 1	Ctrl Medium 1
Day 2	Ctrl Medium 1	Ctrl Medium 1	TNFα	TNFα
Day 3	Ctrl Medium 2	PEMF (ON)	Ctrl Medium 2	PEMF (ON)

**Table 3:** Stimulation groups and schedule for Study 3. Ctrl Medium 1 = 10% fetal bovine serum supplemented growth medium. Ctrl Medium 2 = 1% fetal bovine serum supplemented growth medium.

	Group 1	Group 2	Group 3	Group 4
Day 1	Ctrl Medium 1	Ctrl Medium 1	Ctrl Medium 1	Ctrl Medium 1
Day 2	Ctrl Medium 2	PEMF (ON)	Ctrl Medium 2	PEMF (ON)
Day 3	Ctrl Medium 2	PEMF (ON)	Ctrl Medium 2	PEMF (ON)
Day 4	Ctrl Medium 2	PEMF (ON)	Ctrl Medium 2	PEMF (ON)
Day 5	-	-	Ctrl Medium 2	PEMF (ON)
Day 6	-	-	Ctrl Medium 2	PEMF (ON)
Day 7	-	-	Ctrl Medium 2	PEMF (ON)

**Table 4:** Oligonucleotides used for qPCR analysis.

Primer	Company	Sequence	Assay ID	Amplicon Length (bp)
GAPDH	ABI	*	Hs99999905_m1	122
CD55	ABI	*	Hs00167090_m1	62
CD59	ABI	*	Hs00174141_m1	70
C4bpα	ABI	*	Hs00426339_m1	105
C4bpβ	ABI	*	Hs01103672_m1	74
CFH	ABI	*	Hs00962373_m1	72
CFI	ABI	*	Hs00989715_m1	75
TNFα	ABI	*	Hs00174128_m1	80
IL-6	ABI	*	Hs00174131_m1	95

*: Sequence not provided by the company. ABI (Applied Biosystems, Foster City, CA, USA).

**Table 5:** Antibodies and dyes used.

Specificity and Species	Company	Catalog Number	Stock Concentration	Used Dilution
Goat anti-human CD55	R&D systems, Minneapolis, MN, USA	AF2009	200 µg/mL	1:50
Mouse anti-human CD59	Bio-Rad, Bio-Rad, Feldkirchen, Germany	MCA1054	1 mg/mL	1:50
Mouse anti-human Ki67	Chemicon International Inc., Temecula, CA, USA	MAB4190	1 mg/mL	1:50
Donkey anti-mouse- Alexa Fluor 488	Invitrogen, Waltham, MA, USA	A21202	2 mg/mL	1:200
Donkey anti-goat- cyanine (Cy)3	Dianova, Hamburg, Germany	705-165-147	1.5 mg/mL	1:200
Donkey anti-mouse-Cy3	Dianova, Hamburg, Germany	715-166-150	1.5 mg/mL	1:200

## Data Availability

Supporting data can be obtained from the authors on request.

## Supplementary Materials

The following supporting information can be downloaded at: https://www.mdpi.com/article/10.3390/jpm14070701/s1.
