# Supplementary material for: In Vitro Investigation of Pulsed Electromagnetic Field Stimulation (PEMF) with MAGCELL® ARTHRO on the Regulatory Expression of Soluble and Membrane-Bound Complement Factors and Inflammatory Cytokines in Immortalized Synovial Fibroblasts
Source: J Pers Med. 2024 Jun 29;14(7):701. doi: 10.3390/jpm14070701 (PMC11277808; doi:10.3390/jpm14070701)
Supplement: Supplementary file 1 [file jpm-14-00701-s001.zip › jpm-3049954-supplementary.pdf]

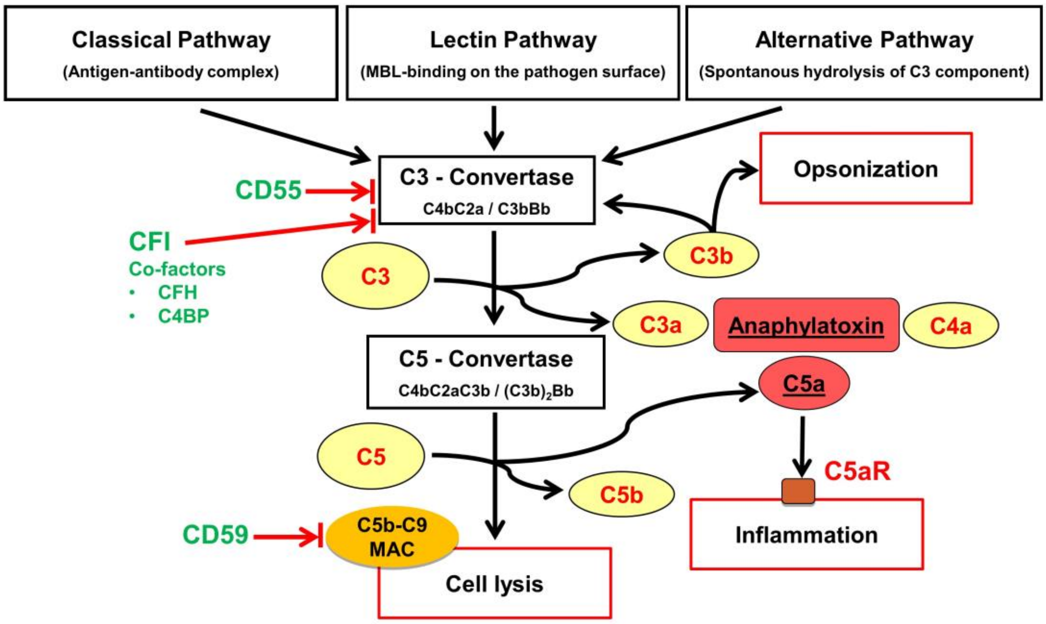

Simplified scheme of the complement activation and regulation processes. C5aR: C5a receptor, MAC: Membrane attack complex, MBL: mannose binding lectin. Image derived and modified from Silawal et al 2021, "Complement Regulation in Human Tenocytes under the Influence of Anaphylatoxin C5a"

1

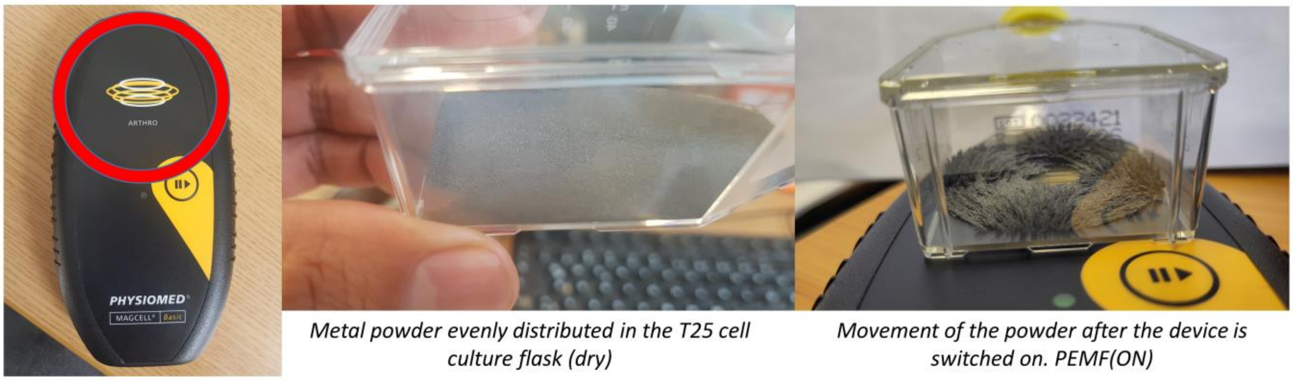

Metal powder evenly distributed in the T25 cell culture flask (dry)

Movement of the powder after the device is switched on. PEMF(ON)

2
